# Persistent Severe Tricuspid Regurgitation After Mitral Transcatheter Edge-to-Edge Repair: Prognostic Implications of Guideline-Based Eligibility for Transcatheter Tricuspid Valve Intervention

**DOI:** 10.1016/j.shj.2026.101051

**Published:** 2026-05-22

**Authors:** Moritz Kühlein, Jule Tervooren, Vera Fortmeier, Amelie Hesse, Elena Rippen, Michelle Fett, Héctor Alfonso Alvarez Covarrubias, Moritz von Scheidt, Ferdinand Roski, Muhammed Gerçek, Shinsuke Yuasa, N. Patrick Mayr, Erion Xhepa, Karl-Ludwig Laugwitz, Michael Joner, Volker Rudolph, Teresa Trenkwalder, Tobias Rheude, Mark Lachmann

**Affiliations:** a Department of Cardiovascular Diseases, German Heart Center Munich, School of Medicine and Health, TUM University Hospital, Technical University of Munich, Munich, Germany; b DZHK (German Center for Cardiovascular Research), Partner Site Munich Heart Alliance, Munich, Germany; c Department of Internal Medicine I, Klinikum Rechts der Isar, TUM University Hospital, School of Medicine and Health, Technical University of Munich, Munich, Germany; d Department of General and Interventional Cardiology, Heart and Diabetes Center Northrhine-Westfalia, Ruhr University Bochum, Bad Oeynhausen, Germany; e Department of Cardiovascular Medicine, Okayama University, Okayama, Japan; f Institute of Anesthesiology, German Heart Center Munich, School of Medicine and Health, TUM University Hospital, Technical University of Munich, Munich, Germany

**Keywords:** Mitral regurgitation, Right heart remodeling, Transcatheter edge-to-edge repair, Transcatheter tricuspid valve intervention, Tricuspid regurgitation

## Abstract

**Background:**

Persistent tricuspid regurgitation (TR) after mitral transcatheter edge-to-edge repair (M-TEER) is common and associated with adverse outcomes. However, the prognostic relevance of persistent TR may differ depending on anatomical suitability for staged transcatheter tricuspid valve intervention (TTVI). This study aimed to characterize the prevalence, determinants, and prognostic significance of persistent severe TR after M-TEER, with a particular focus on TTVI eligibility.

**Methods:**

In this dual-center retrospective cohort study, 905 patients undergoing M-TEER for severe mitral regurgitation between 2015 and 2025 were analyzed. TR severity was assessed at baseline and at 3-month follow-up. Patients with persistent severe TR were categorized as TTVI eligible or TTVI ineligible according to current guideline-based criteria. Multivariable logistic regression identified factors associated with persistent TR, and survival analyses evaluated the prognostic impact of TR persistence.

**Results:**

Among 905 patients (median age 79.7 years; 43.4% female), 1-year and 2-year survival rates after M-TEER were 87.7% (95% CI: 85.6%–89.9%) and 77.6% (95% CI: 74.9%–80.4%), respectively. Follow-up echocardiography was available in 454 patients (50.2%). Persistent severe TR was present in 16.3% at 3 months. TTVI-ineligible patients had markedly reduced 2-year survival (39.5%; 95% CI: 22.8%–68.4%), whereas TTVI-eligible patients showed survival similar to those without persistent TR (82.9% [95% CI: 72.8%–94.5%] vs. 87.7% [95% CI: 84.4%–91.2%]). In multivariable analysis, TTVI ineligibility was independently associated with elevated 2-year mortality (hazard ratio [HR]: 5.25 [95% CI: 1.39–19.8]). Advanced right heart remodeling and higher baseline TR severity were strongly associated with TR persistence. Moreover, female sex was independently associated with persistent TR despite similar mitral regurgitation etiology and baseline TR severity between sexes.

**Conclusion:**

Persistent TR after M-TEER identifies a high-risk subgroup, but adverse outcomes are primarily driven by patients ineligible for TTVI. Early anticipation of TR persistence, particularly in women, and structured assessment of TTVI eligibility may enable timely intervention and prevent progression to untreatable right-sided heart failure.

## Highlights

• Persistent TR after M-TEER is common and has been associated with adverse outcomes. However, prior studies have not differentiated prognosis based on guideline-directed eligibility for TTVI. The determinants of TR persistence, including potential sex-specific differences, remain incompletely understood.
• Persistent severe TR at 3 months occurs in ∼16% of patients following M-TEER, but adverse outcomes are driven primarily by those ineligible for TTVI. Female sex, right heart remodeling, and higher baseline TR grade were independently associated with TR persistence. Importantly, patients eligible for TTVI show survival comparable to those without persistent TR.
• Prospective studies are needed to evaluate whether earlier identification of TR persistence and timely staged TTVI can improve long-term outcomes. Moreover, sex-specific mechanisms of right heart remodeling warrant further investigation to optimize care in women. Finally, clinical algorithms integrating early assessment of TR persistence after M-TEER and structured TTVI evaluation may help prevent progression to end-stage, treatment-limited right-sided heart failure.

## Graphical Abstract

Graphical Abstract: Persistent TR after M-TEER identifies a high-risk subgroup, with adverse outcomes primarily driven by patients considered as ineligible for TTVI according to guideline recommendations.

**Figure:**
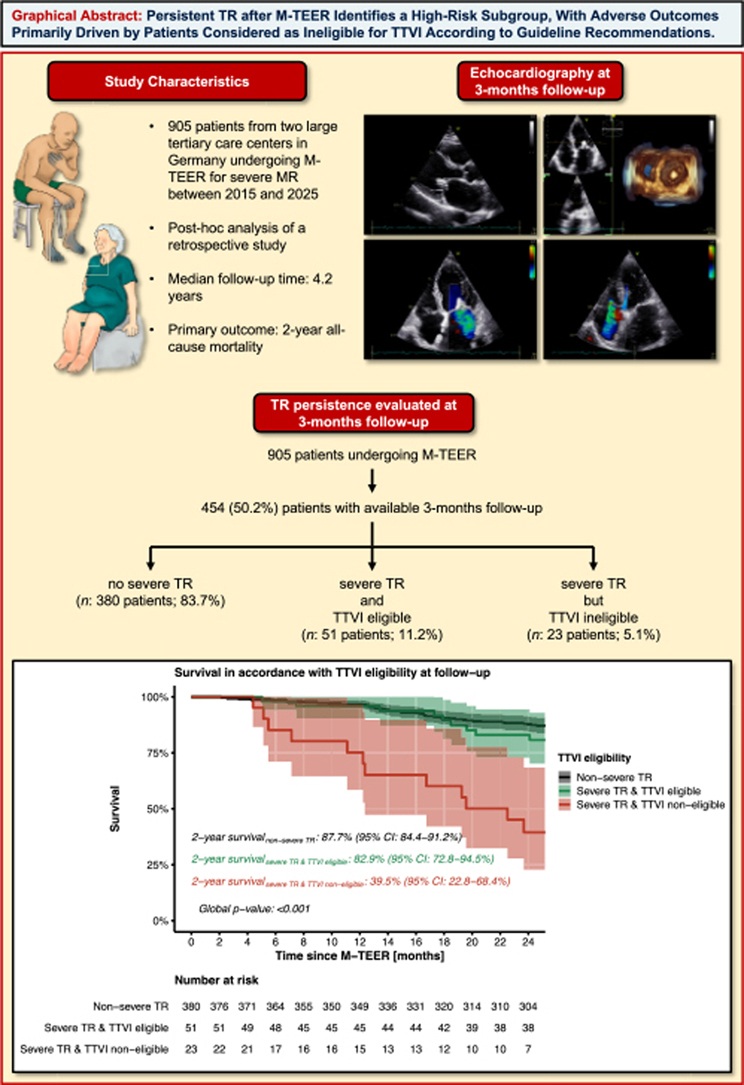

## Introduction

Tricuspid regurgitation (TR) frequently persists in patients treated for mitral regurgitation (MR).^1^, ^2^, ^3^ Mechanistically, TR may result from progressive right ventricular (RV) remodeling due to longstanding pulmonary hypertension with subsequent papillary muscle displacement and leaflet tethering (secondary “ventricular” TR), or it may develop in the setting of atrial fibrillation with biatrial dilatation and leaflet malcoaptation (secondary “atrial” TR).^4^ Both mechanisms may continue despite successful correction of the index MR, causing persistence or even contributing to progression of TR. A growing body of evidence, including analyses based on unsupervised machine learning, indicates that persistent severe or massive TR after mitral transcatheter edge-to-edge repair (M-TEER) is associated with increased mortality, underscoring its prognostic impact.^5^

Over recent years, the perception of TR has shifted from that of a “forgotten valve” to a distinct therapeutic target. Randomized evidence now supports transcatheter therapies that directly address TR. The TRILUMINATE Pivotal trial demonstrated that tricuspid valve transcatheter edge-to-edge repair safely reduced TR severity and improved quality of life compared with medical therapy, with durable reductions through 2 years and fewer recurrent heart failure hospitalizations at longer follow-up.^6^^,^^7^ Similarly, the TRISCEND II trial showed that transcatheter tricuspid valve replacement was superior to medical therapy alone for the primary composite endpoint, driven primarily by symptomatic and quality-of-life improvements.^8^ Reflecting this maturation of evidence, regulatory approvals in the United States now include both a repair system (TriClip) and a replacement system (EVOQUE), further accelerating clinical adoption. The 2025 ESC/EACTS guidelines for the management of valvular heart disease accordingly provide a Class IIa recommendation for transcatheter treatment of severe, symptomatic TR in appropriately selected patients.^9^

In the context of M-TEER, where severe TR frequently persists despite successful left-sided intervention, the proportion of these patients who would subsequently qualify for transcatheter tricuspid valve intervention (TTVI) according to current guideline-based selection criteria remains unclear. Identifying this subgroup is clinically important, as it could shift management from a “fire-and-forget” approach—where residual TR is often underestimated, and patients may be lost to follow-up—to a proactive strategy that recognizes patients at ongoing risk and facilitates closer surveillance as well as timely referral for targeted TTVI.

To address this gap, the present study aimed to accomplish the following:

1. Assess the persistence of severe TR after M-TEER and identify clinical and echocardiographic factors associated with TR persistence,
2. Determine the prevalence of TTVI eligibility among patients with persistent severe TR following M-TEER, and
3. Characterize TTVI-eligible versus ineligible patients and their associated long-term outcomes in this high-risk cohort.

## Methods

### Study Population

This is a *post hoc* analysis of prospectively and systematically collected data from patients who underwent M-TEER for symptomatic moderate-to-severe or severe MR at 2 high-volume tertiary care centers in Germany between 2015 and 2025 (Heart and Diabetes Center Northrhine-Westfalia and German Heart Center Munich). Baseline demographic and clinical characteristics were obtained from registry data or clinical records. All patients underwent transthoracic as well as transesophageal echocardiography prior to M-TEER. Moreover, clinical follow-up, including transthoracic echocardiography, was routinely performed at 3 months after M-TEER. The local ethics committees at each participating center approved the collection and analysis of data in accordance with the Declaration of Helsinki, and all patients gave their written informed consent to be enrolled in an observational registry.

### Transthoracic Echocardiography

All echocardiographic studies were performed by experienced institutional cardiologists during clinical routine using commercially available equipment. Echocardiographic measures were assessed according to the current guideline recommendations,^10^ and classification of MR severity was performed using an integrative, multiparametric 4-grade approach (mild ≙ I°, moderate ≙ II°, severe ≙ III°, and massive ≙ IV°). Intermediate grades (e.g., II°+) were used in clinical practice to denote moderate-to-severe MR—that is, more than moderate but not fulfilling criteria for severe MR—and were considered consistent with the inclusion criteria for M-TEER. In addition, the etiology of MR was defined as either primary (mitral valve [MV] prolapse ± flail leaflet), secondary (ventricular dilatation, atrial dilatation, or both), or mixed. Tricuspid annular plane systolic excursion (TAPSE) was assessed in an apical 4-chamber echocardiographic view by placing the M-mode cursor at the lateral tricuspid valve annulus and measuring the movement of the tricuspid annulus toward the apex during systole. Systolic pulmonary artery pressure (sPAP) was calculated by adding the peak transvalvular gradient across the tricuspid valve (estimated from the continuous wave Doppler profile of the TR jet) to the right atrial pressure. As outlined in contemporary guidelines, the latter was inferred from the diameter and collapsibility of the inferior vena cava.^10^^,^^11^

### Transcatheter Edge-to-Edge Repair

In all patients, the indication for M-TEER was symptomatic moderate-to-severe or severe MR, and the local heart teams approved all procedures. Procedures were performed under general anesthesia with 3-dimensional transesophageal echocardiographic and fluoroscopic guidance. The number of devices implanted was determined by the discretion of the treating physician. Details of the procedures have been described previously.^12^

### TTVI Eligibility Criteria

At follow-up, all patients with persistent or newly developed severe TR were systematically evaluated for potential eligibility for TTVI. Eligibility was assessed according to key anatomic and hemodynamic parameters consistent with the 2025 European Society of Cardiology (ESC)/European Association for Cardio-Thoracic Surgery (EACTS) guidelines for the Management of Valvular Heart Disease,^9^ reflecting contemporary standards for TTVI candidate selection.

Patients were classified as TTVI eligible if they met all of the following criteria at follow-up:

• Severe or greater TR (TR grade ≥ III),
• sPAP < 70 mmHg (indicating absence of severe pulmonary hypertension),
• No persistent severe MR (MR < grade III),
• Left ventricular ejection fraction (LVEF) > 30% (indicating absence of severe LV dysfunction), and
• TAPSE > 10 mm (indicating absence of severe RV dysfunction).

Patients who failed to meet 1 or more of these criteria were classified as TTVI ineligible. Accordingly, 3 groups were defined for subsequent analyses:

1. No severe TR at follow-up,
2. TTVI eligible, and
3. TTVI ineligible (defined by the presence of any exclusion criterion).

### Clinical Endpoint Definition

Given that the present study population consisted predominantly of elderly and multimorbid patients, postprocedural 2-year all-cause mortality was selected as the primary clinical endpoint. This follow-up duration was chosen to provide a balanced perspective: it is long enough to capture meaningful outcome differences yet short enough to minimize the confounding influence of non-cardiac causes of death that become more prevalent with extended observation. In order to ensure complete follow-up for all participants, vital status was systematically verified through the German Civil Registry. For patients residing outside Germany, survival data were additionally obtained from treating physicians, general practitioners, and referring hospitals.

### Statistical Analysis

All statistical analyses were performed in R (R version 4.3.2; R Foundation for Statistical Computing, Vienna, Austria).

Categorical data are presented as counts and frequencies (%), while continuous data are expressed as median and interquartile range (IQR). Chi-square or Fisher’s exact tests were used to evaluate the association between categorical variables, and independent-samples Wilcoxon tests were used to compare continuous variables.

Pairwise comparisons of preprocedural and postprocedural data were performed using a paired-samples Wilcoxon test.

Univariable logistic regression analyses were conducted to identify clinical, laboratory, and echocardiographic factors associated with persistent TR after M-TEER. Odds ratios (ORs) and 95% CIs were calculated for each variable. To ensure model stability and reduce the risk of overfitting given the limited number of events and complete cases, only variables demonstrating a univariable association (*p* value ≤0.05) were included in the multivariable model. Multicollinearity among covariates was assessed using variance inflation factors.

Finally, survival was illustrated using the Kaplan-Meier method, and the log-rank test was applied to compare survival rates. Moreover, Cox proportional hazards regression analysis was performed to evaluate the association between TTVI eligibility and 2-year all-cause mortality among patients with severe TR at follow-up. The multivariable model included TTVI eligibility status, age at follow-up, LVEF, sPAP, and TAPSE, selected based on clinical relevance. The proportional hazards assumption for Cox regression was tested using Schoenfeld residuals.

A *p* value of ≤0.05 was considered to indicate statistical significance.

## Results

### Clinical Characteristics of the Study Population

A total of 905 patients who underwent M-TEER for moderate-to-severe and severe MR between 2015 and 2025 were included in this dual-center analysis (525 patients [58.0%] treated in Munich and 380 patients [42.0%] treated in Bad Oeynhausen, Germany). Baseline demographic, clinical, and echocardiographic data are summarized in Tables 1 and 2. The median age of the study population was 79.7 years (IQR: 74.2–83.4 years), and 56.6% of patients were male (Table 1). The majority of patients experienced severe exertional dyspnea, with 67.3% in New York Heart Association (NYHA) functional class III and 13.3% in class IV. Additionally, patients presented with a median N-terminal prohormone of brain natriuretic peptide (NT-proBNP) level of 2810 pg/mL (IQR: 1293–6545 pg/mL). In this all-comers registry, MR was classified as primary in 307 patients (33.9%), secondary in 497 patients (54.9%), and mixed in 101 patients (11.2%). A reduction in MR severity to ≤ II/IV° was achieved in 853 of the 905 patients (94.3%), with a postprocedural median MV gradient of 3.0 mmHg (IQR: 2.0–4.0 mmHg). The number of implanted devices per procedure varied, with 1 clip implanted in 571 patients (63.1%), 2 clips in 308 patients (34.0%), and 3 clips in 26 patients (2.9%). An overview of implanted device types is provided in Supplementary Table 1: MitraClip systems were used in 663 patients (73.3%), whereas PASCAL devices were used in 242 patients (26.7%).

**Table 1:** Demographic and clinical baseline characteristics of the study population

	Study population (*n* = 905 patients)
Age, y	79.7 (74.2–83.4)
Female, no. (%)	393 (43.4%)
BMI, kg/m^2^	25.0 (22.5–28.2)
Arterial hypertension, no. (%)	754 (83.3%)
Diabetes mellitus, no. (%)	231 (25.5%)
History of CAD, no. (%)	555 (61.3%)
History of COPD, no. (%)	143 (15.8%)
History of atrial fibrillation, no. (%)	657 (72.6%)
NYHA ≤ II, no. (%)	176 (19.4%)
NYHA III, no. (%)	609 (67.3%)
NYHA IV, no. (%)	120 (13.3%)
EuroSCORE II, %	5.13 (3.26–8.54)
eGFR, mL/min	48 (35-62)
NT-proBNP, pg/mL	2810 (1293–6545)
Hemoglobin, g/dL	12.6 (11.1–13.7)
Dialysis, no. (%)	36 (4.0%)
Etiology	
Primary, no. (%)	307 (33.9%)
Secondary, no. (%)	497 (54.9%)
Mixed, no. (%)	101 (11.2%)

Categorical data are presented as counts and frequencies (%), while continuous data are expressed as median and interquartile range.

Abbreviations: BMI, body mass index; CAD, coronary artery disease; COPD, chronic obstructive pulmonary disease; eGFR, estimated glomerular filtration rate; EuroSCORE II, European System for Cardiac Operative Risk Evaluation II (a refined risk model used to estimate the mortality risk for patients undergoing cardiac surgery); NT-proBNP, N-terminal prohormone of brain natriuretic peptide; NYHA, New York Heart Association.

**Table 2:** Echocardiographic characteristics of the study population at baseline and follow-up

	Study population		*p* value
	Baseline (*n* = 905 patients)	Follow-up (*n* = 454 patients)	
LVEF, %	48 (33–58)	50 (34–57)	0.005
LVEDD, mm	57 (50–63)	54 (48–62)	<0.001
LVESD, mm	41 (34–51)	40 (33–52)	0.411
LVEDV, mL	144 (95–196)	125 (88–186)	0.093
LVESV, mL	80 (44–128)	67 (41–109)	0.558
MV EROA, cm^2^	0.3 (0.2–0.4)		
MR vena contract width, cm	0.7 (0.6–1.0)		
MV regurgitation volume, mL	47 (35–66)		
MV gradient, mmHg		3 (2–4)	
LA volume, mL	125 (96–169)	107 (79–146)	<0.001
sPAP, mmHg	46 (36–58)	41 (32–54)	0.021
Right midventricular diameter, mm	31 (27–35)	31 (26–35)	0.062
TAPSE, mm	17 (14–20)	18 (15–21)	0.367
RA area, cm^2^	26 (20–31)	23 (18–30)	0.874
MR II and II+/IV°, No. (%)∗	485 (53.6%)	419 (92.3%)	<0.001
MR III and III+/IV°, No. (%)∗	246 (27.2%)	32 (7.0%)	<0.001
MR IV/IV°, No. (%)∗	174 (19.2%)	3 (0.7%)	<0.001
TR ≥ III/IV°, No. (%)	209 (23.2%)	74 (16.3%)	<0.001

Categorical data are presented as counts and frequencies (%), while continuous data are expressed as median and interquartile range.

Abbreviations: LA volume, left atrial volume; LVEDD, left ventricular end-diastolic diameter; LVEDV, left ventricular end-diastolic volume; LVEF, left ventricular ejection fraction; LVESD, left ventricular end-systolic diameter; LVESV, left ventricular end-systolic volume; MR, mitral regurgitation; MV EROA, mitral valve effective regurgitant orifice area; MV gradient, mitral valve gradient; MV regurgitation volume, mitral valve regurgitation volume; RA area, right atrial area; sPAP, systolic pulmonary artery pressure; TAPSE, tricuspid annular plane systolic excursion; TR, tricuspid regurgitation.

∗ MR at follow-up ≤ I, II and II+/IV°.

**Figure 1:**
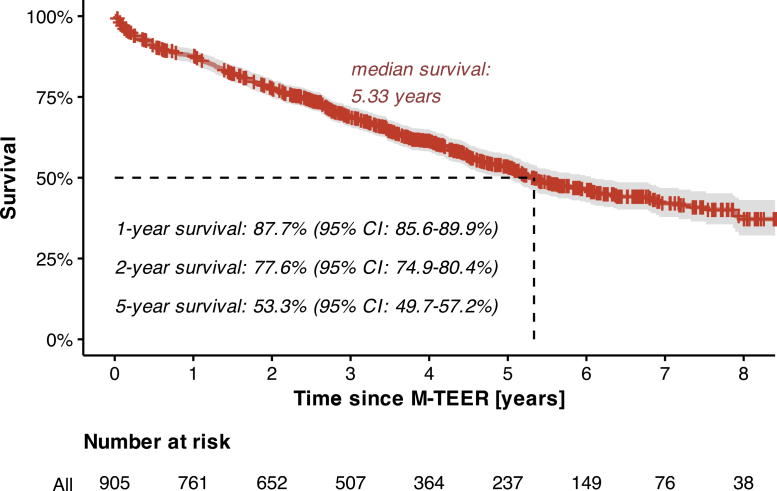
**Survival plot for all patients enrolled in this dual-center registry of patients who underwentM-TEERbetween 2015 and 2025.** Abbreviation: M-TEER, mitral transcatheter edge-to-edge repair.

During the study period, 404 deaths were documented among the enrolled patients. The remaining participants were followed for a median duration of 4.31 years (IQR: 2.89–6.05 years). The overall 1-year and 2-year survival rates following M-TEER were 87.7% (95% CI: 85.6%–89.9%) and 77.6% (95% CI: 74.9%–80.4%), respectively (Figure 1).

Follow-up echocardiography was available for 454 patients (50.2%), typically performed at a median of 100 days (IQR: 84–134 days) after the M-TEER procedure. Importantly, patients who did not attend follow-up echocardiography, including those with missing or unrecorded data, had a significantly higher risk of 2-year mortality compared to those who did attend (hazard ratio [HR]: 2.31 [95% CI: 1.72–3.12], *p* value: <0.001) (Supplementary Figure 1). Supplementary Tables 2 and 3 provide a detailed comparison of baseline characteristics between patients with and without available follow-up echocardiography. Patients without follow-up were more clinically decompensated at baseline, as reflected by higher NT-proBNP levels (3350 pg/mL [IQR: 1525–7628 pg/mL] vs. 2555 pg/mL [IQR: 1160–5510 pg/mL]) and a greater proportion presenting in NYHA functional class IV (17.5% vs. 9.0%). In addition, LVEF was lower (46% [IQR: 31%–58%) vs. 50% [IQR: 35%–58%]), and sPAP was higher in patients not attending follow-up (47 mmHg [IQR: 37–60 mmHg] vs. 45 mmHg [IQR: 34–56 mmHg]). In contrast, RV function (as assessed by TAPSE) as well as the proportion of patients with severe TR at baseline were comparable between groups.

### Amelioration of Pulmonary Artery Pressure After M-TEER Is Associated With Improvement in TR Severity

At follow-up, successful reduction of MR severity after M-TEER was accompanied by favorable left-sided and pulmonary hemodynamic changes (Table 2). Modest but statistically significant improvements were observed in left ventricular function and dimensions, including increases in LVEF (from 48% [95% CI: 33%–58%] at baseline to 50% [IQR: 34–57] at follow-up, *p* value: 0.005) and reductions in left ventricular end-diastolic diameter (from 57 mm [IQR: 50–63 mm] at baseline to 54 mm [IQR: 48–62 mm] at follow-up, *p* value: <0.001). Moreover, left atrial volume decreased significantly from 125 mL (IQR: 96–169 mL) at baseline to 107 mL (IQR: 79–146 mL) at follow-up (*p* value: <0.001), and sPAP declined from 46 mm Hg (IQR: 36–58 mmHg) to 41 mmHg (IQR: 32–54 mm Hg; *p* value: 0.021).

Despite these left-sided and pulmonary improvements, RV function (assessed by TAPSE) remained largely unchanged (17 mm [IQR: 14–20 mm] at baseline vs. 18 mm [IQR: 15–21 mm] at follow-up; *p* value: 0.367), and no consistent reverse remodeling of the right ventricle or right atrium was observed.

**Figure 2:**
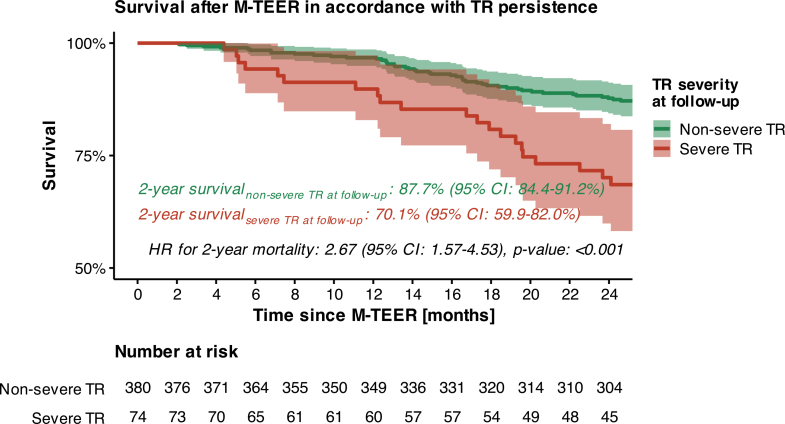
**Survival stratified by persistence of severe TR at 3 months follow-up after M-TEER.** Abbreviations: HR, hazard ratio; M-TEER, mitral transcatheter edge-to-edge repair; TR, tricuspid regurgitation.

Concordant with the decrease in pulmonary artery pressure, the overall prevalence of severe TR was reduced from 23.2% at baseline to 16.3% at follow-up (*p* value: <0.001). Importantly, patients with persistent or newly developed severe TR after M-TEER exhibited a markedly higher 2-year mortality compared to those without severe TR (HR: 2.67; 95% CI: 1.57–4.53; *p* value: <0.001) (Figure 2).

### Female Sex, Right Heart Remodeling, and Pre-Existing TR Are Associated With Persistent Severe TR After M-TEER

In univariable logistic regression analysis, several baseline characteristics were associated with the persistence of severe TR at follow-up (Table 3). Older age (OR: 1.07 per year [95% CI: 1.03–1.12]; *p* value: 0.001), female sex (OR: 1.75 [95% CI: 1.06–2.90]; *p* value: 0.030), atrial fibrillation (OR: 5.29 [95% CI: 2.42–14.0]; *p* value: <0.001), RV enlargement (OR: 1.12 per mm [95% CI: 1.07–1.18]; *p* value: <0.001), right atrial enlargement (OR: 1.09 per cm^2^ [95% CI: 1.06–1.13]; *p* value: <0.001), and greater preprocedural TR grade (OR: 6.83 [95% CI: 4.52–10.8]; *p* value: <0.001) were all significantly associated with an increased likelihood of persistent severe TR.

**Table 3:** Univariable and multivariable logistic regression analysis of clinical, laboratory, and echocardiographic factors associated with persistence of severe TR following M-TEER

	Univariable analysis		Multivariable analysis	
	OR (95% CI)	*p* value	OR (95% CI)	*P* value
Age (per 1-y increment)	1.07 (1.03–1.12)	<0.001	1.07 (0.98–1.20)	0.160
Female sex	1.75 (1.06–2.90)	0.030	10.1 (2.37–57.4)	0.004
BMI (per 1 kg/m^2^ increment)	1.02 (0.97–1.07)	0.348		
Arterial hypertension	2.12 (0.95–5.68)	0.093		
History of CAD	0.42 (0.25–0.70)	0.001	0.66 (0.21–2.09)	0.479
History of COPD	1.26 (0.63–2.38)	0.496		
History of atrial fibrillation	5.29 (2.42–14.0)	<0.001	0.48 (0.09–2.99)	0.408
NYHA class (per grade severity increment)	1.41 (0.88–2.30)	0.159		
eGFR (per 1 mL/min increment)	0.98 (0.97–1.00)	0.011	0.95 (0.91–0.98)	0.006
NT-proBNP (per 1000 pg/mL increment)	0.99 (0.94–1.03)	0.629		
LVEF (per 1% increment)	1.03 (1.01–1.05)	0.001	1.06 (1.01–1.11)	0.022
Preprocedural MR severity (per grade severity increment)	1.29 (0.94–1.78)	0.116		
LA volume (per 1 mL increment)	1.00 (1.00–1.01)	0.128		
sPAP (per 1 mmHg increment)	0.99 (0.98–1.01)	0.347		
Right midventricular diameter (per 1 mm increment)	1.12 (1.07–1.18)	<0.001	1.18 (1.06–1.32)	0.003
TAPSE (per 1 mm increment)	0.99 (0.94–1.04)	0.741		
RA area (per 1 cm^2^ increment)	1.09 (1.06–1.13)	<0.001	1.17 (1.07–1.29)	0.001
Preprocedural TR severity (per grade severity increment)	6.83 (4.52–10.9)	<0.001	7.59 (3.49–20.0)	<0.001
Postprocedural MR severity (per grade severity increment)	1.22 (0.89–1.66)	0.202		
Postprocedural MV gradient (per 1 mmHg increment)	0.88 (0.73–1.06)	0.188		
Primary MR etiology	0.67 (0.37–1.16)	0.167		
Secondary MR etiology	1.33 (0.80–2.24)	0.275		
Mixed MR etiology	1.14 (0.50–2.37)	0.730		

Abbreviations: BMI, body mass index; CAD, coronary artery disease; COPD, chronic obstructive pulmonary disease; eGFR, estimated glomerular filtration rate; LA volume, left atrial volume; LVEF, left ventricular ejection fraction; MR, mitral regurgitation; M-TEER, mitral transcatheter edge-to-edge repair; MV, mitral valve; NT-proBNP, N-terminal prohormone of brain natriuretic peptide; NYHA, New York Heart Association; OR, odds ratio; RA area, right atrial area; sPAP, systolic pulmonary artery pressure; TAPSE, tricuspid annular plane systolic excursion; TR, tricuspid regurgitation.

In addition, impaired renal function (estimated glomerular filtration rate: OR: 0.98 per mL/min [95% CI: 0.97–1.00]; *p* value: 0.011) and higher LVEF (OR: 1.03 per 1% increase [95% CI: 1.01–1.05]; *p* value: 0.001) were associated with TR persistence, whereas a history of coronary artery disease was associated with a lower likelihood of persistent TR (OR: 0.42 [95% CI: 0.25–0.70]; *p* value: 0.001).

In multivariable analysis, female sex (adjusted OR: 10.1 [95% CI: 2.37–57.4]; *p* value: 0.004), impaired renal function (estimated glomerular filtration rate: adjusted OR: 0.95 per mL/min [95% CI: 0.91–0.98]; *p* value: 0.006), higher LVEF (adjusted OR: 1.06 per 1% increase [95% CI: 1.01–1.11]; *p* value: 0.022), RV enlargement (adjusted OR: 1.18 per mm [95% CI: 1.06–1.32]; *p* value: 0.003), right atrial enlargement (adjusted OR: 1.17 per cm^2^ [95% CI: 1.07–1.29]; *p* value: 0.001), and greater preprocedural TR severity (adjusted OR: 7.59 [95% CI: 3.49–20.0]; *p* value: <0.001) remained independently associated with persistence of severe TR at follow-up.

Assessment of multicollinearity showed no evidence of relevant multicollinearity among covariates used in the multivariable analysis (variance inflation factors <2.2).

Moreover, in an additional exploratory multivariable logistic regression analysis including both device type and treatment center, neither device type (MitraClip vs. PASCAL; *p* value: 0.238) nor treatment center (Munich vs. Bad Oeynhausen; *p* value: 0.358) was significantly associated with persistence of severe TR.

### Eligibility for Transcatheter Tricuspid Valve Intervention and Associated Outcomes

Among the 74 patients with severe TR at follow-up, most remained symptomatic, with 37.8% in NYHA functional class II and 50.0% in class III. Evidence of persistent systemic congestion was reflected by markedly elevated NT-proBNP levels (median 2720 pg/mL [95% CI: 1598–5038 pg/mL]) (Tables 4 and 5).

**Table 4:** Comparison of baseline demographic and clinical characteristics among patients with persistent severe TR at follow-up after M-TEER

	Study population			*p* value
	All (*n* = 74 patients)	Eligible (*n* = 51 patients)	Ineligible (*n* = 23 patients)	
Age at follow-up, y	82.0 (78.9–84.6)	82.1 (79.5–84.7)	80.6 (76.8–84.4)	0.141
Female, no. (%)	41 (55.4%)	26 (51.0%)	15 (65.2%)	0.375
BMI, kg/m^2^	25.0 (22.5–29.1)	26.1 (23.5–30.0)	22.9 (21.3–26.4)	0.014
Arterial hypertension, no. (%)	68 (91.9%)	48 (94.1%)	20 (87.0%)	0.367
Diabetes mellitus, no. (%)	21 (28.4%)	13 (25.5%)	8 (34.8%)	0.588
History of CAD, no. (%)	31 (41.9%)	21 (41.2%)	10 (43.5%)	1.0
History of COPD, no. (%)	13 (17.6%)	9 (17.6%)	4 (17.4%)	1.0
History of atrial fibrillation, no. (%)	68 (91.9%)	48 (94.1%)	20 (87.0%)	0.367
NYHA I at follow-up, no. (%)	9 (12.2%)	6 (11.8%)	3 (13.0%)	1.0
NYHA II at follow-up, no. (%)	28 (37.8%)	24 (47.1%)	4 (17.4%)	0.030
NYHA III at follow-up, no. (%)	37 (50.0%)	21 (41.8%)	16 (69.6%)	0.045
NYHA IV at follow-up, no. (%)	0	0	0	/
EuroSCORE II, %	5.4 (3.7–8.5)	5.5 (4.2–7.7)	4.8 (3.5–11.2)	0.952
eGFR, mL/min	43 (30.3–53.8)	46 (36–55)	42 (24–54)	0.347
NT-proBNP, pg/mL	2720 (1598-5038)	2360 (1228-3500)	4765 (2652–7230)	0.006
Hemoglobin, g/dL	12.4 (10.5–13.4)	12.7 (11.6–13.6)	10.8 (9.9–12.8)	0.007
Dialysis, no. (%)	2 (2.7%)	1 (2.0%)	1 (4.3%)	0.528
Etiology				
Primary, no. (%)	19 (25.7%)	14 (27.5%)	5 (21.7%)	0.816
Secondary, no. (%)	46 (62.2%)	32 (62.7%)	14 (60.9%)	1.0
Mixed, no. (%)	9 (12.2%)	5 (9.8%)	4 (17.4%)	0.446

Categorical data are presented as counts and frequencies (%), while continuous data are expressed as median and interquartile range.

Abbreviations: BMI, body mass index; CAD, coronary artery disease; COPD, chronic obstructive pulmonary disease; eGFR, estimated glomerular filtration rate; EuroSCORE II, European System for Cardiac Operative Risk Evaluation II (a refined risk model used to estimate the mortality risk for patients undergoing cardiac surgery); M-TEER, mitral transcatheter edge-to-edge repair; NT-proBNP, N-terminal prohormone of brain natriuretic peptide; NYHA, New York Heart Association; TR, tricuspid regurgitation.

**Table 5:** Comparison of follow-up echocardiographic characteristics among patients with persistent severe TR at follow-up after M-TEER

	Study population			*p* value
	All (*n* = 74 patients)	Eligible (*n* = 51 patients)	Ineligible (*n* = 23 patients)	
LVEF, %	52 (45–59)	54 (47–60)	45 (30–51)	0.006
LVEDD, mm	47 (44–54)	46 (43–52)	51 (45–56)	0.081
LVESD, mm	34 (30–43)	32 (30–37)	42 (34–49)	0.038
LVEDV, mL	101 (73–139)	94 (73–133)	106 (93–186)	0.218
LVESV, mL	55 (30–78)	49 (30–67)	64 (41–100)	0.134
MV gradient, mmHg	3 (2–5)	3 (2–5)	3 (3–4)	0.789
LA volume, mL	118 (86–143)	120 (83–141)	113 (89–148)	0.631
sPAP, mmHg	49 (38–57)	49 (39–56)	48 (38–69)	0.438
Right midventricular diameter, mm	33 (28–40)	34 (30–40)	31 (28–40)	0.549
TAPSE, mm	17 (14–21)	17 (15–22)	16 (11–19)	0.047
TAPSE/sPAP, mm/mmHg	0.350 (0.272–0.465)	0.386 (0.295–0.485)	0.267 (0.181–0.355)	0.001
RA area, cm^2^	32 (25–39)	33 (26–39)	27 (23–40)	0.423
MR II and II+/IV°, No. (%)	70 (94.6%)	51 (100%)	19 (82.6%)	0.008
MR III and III+/IV°, No. (%)	4 (5.4%)	0	4 (17.4%)	0.008
MR IV/IV°, No. (%)	0	0	0	/
TR III/IV°, No. (%)	55 (74.3%)	39 (76.5%)	16 (69.6%)	0.732
TR IV/IV°, No. (%)	19 (25.7%)	12 (23.5%)	7 (30.4%)	0.732

Categorical data are presented as counts and frequencies (%), while continuous data are expressed as median and interquartile range.

Abbreviations: LA volume, left atrial volume; LVEDD, left ventricular end-diastolic diameter; LVEDV, left ventricular end-diastolic volume; LVEF, left ventricular ejection fraction; LVESD, left ventricular end-systolic diameter; LVESV, left ventricular end-systolic volume; MR, mitral regurgitation; M-TEER, mitral transcatheter edge-to-edge repair; MV gradient, mitral valve gradient; RA area, right atrial area; sPAP, systolic pulmonary artery pressure; TAPSE, tricuspid annular plane systolic excursion; TR, tricuspid regurgitation.

Of these 74 patients, 51 (68.9%) fulfilled TTVI eligibility criteria. The remaining 23 patients (31.1%) were deemed ineligible, most frequently due to severe pulmonary hypertension (defined as sPAP >70 mmHg; 26.1%), severe left ventricular dysfunction (defined as LVEF <30%; 26.1%), or RV dysfunction (defined as TAPSE <10 mm; 21.7%). Survival at 2 years after M-TEER differed strikingly according to T-TEER eligibility (Graphical Abstract). Patients considered ineligible had a markedly worse prognosis, with a 2-year survival of only 39.5% (95% CI: 22.8%–68.4%) and a 6-fold higher risk of mortality compared to patients without severe TR (HR: 6.79 [95% CI: 3.58–12.88]; *p* value: <0.001). In contrast, eligible patients exhibited 2-year survival rates comparable to those without severe TR (82.9% [95% CI: 72.8%–94.5%] vs. 87.7% [95% CI: 84.4%–91.2%]; HR: 1.40 [95% CI: 0.66-2.97]; *p* value: 0.382). Notably, in a 3-month landmark analysis excluding patients who died within the first 3 months after M-TEER, survival differences according to follow-up TTVI classification remained significant (*p* value: <0.001). At 2 years after the 3-month landmark, survival was 86.7% (95% CI: 83.3%–90.3%) in patients without severe TR, 76.4% (95% CI: 65.1%–89.6%) in TTVI-eligible patients, and 39.5% (95% CI: 22.8%–68.4%) in TTVI-ineligible patients (Supplementary Figure 2).

In an expanded multivariable Cox regression model adjusting for age, left and right ventricular function, and pulmonary artery pressure, T-TEER ineligibility remained the only factor independently associated with 2-year mortality among patients with persistent severe TR after M-TEER (adjusted HR: 5.25 [95% CI: 1.39–19.8]; *p* value: 0.014) (Table 6). None of the other covariates—including age, LVEF, sPAP, or TAPSE—were independently associated with outcome (all *p* value: >0.1).

**Table 6:** Multivariable Cox regression analysis for 2-year mortality among patients with severe TR at follow-up according to TTVI eligibility

	HR (95% CI)	*p* value
Severe TR at follow-up and TTVI ineligibility (vs. TTVI eligibility)	5.25 (1.39–19.8)	0.014
Age at follow-up (per 1-y increment)	0.95 (0.89–1.02)	0.136
LVEF at follow-up (per 1% increment)	1.00 (0.96–1.05)	0.846
sPAP at follow-up (per 1 mmHg increment)	1.00 (0.97–1.02)	0.794
TAPSE at follow-up (per 1 mm increment)	1.01 (0.91–1.12)	0.842

The proportional hazards assumption was confirmed using Schoenfeld residuals and was not violated for any covariate (*p* value for TTVI ineligibility: 0.917; for age: 0.358; for LVEF: 0.267; for sPAP: 0.649; for TAPSE: 0.507) or for the global model (*p* value: 0.643).

Abbreviations: HR, hazard ratio; LVEF, left ventricular ejection fraction; sPAP, systolic pulmonary artery pressure; TAPSE, tricuspid annular plane systolic excursion; TR, tricuspid regurgitation; TTVI, transcatheter tricuspid valve intervention.

Notably, atrial fibrillation was present in 657 of 905 patients overall (72.6%) (Table 1) and in 48 of 51 (94.1%) patients with persistent severe TR who met T-TEER eligibility criteria (Table 4). Moreover, ineligible patients demonstrated significantly poorer left ventricular function (LVEF 45% [IQR: 30%–51%] vs. 54% [IQR: 47%-60%]; *p* value: 0.006), reduced RV performance (TAPSE 16 mm [IQR: 11–19 mm] vs. 17 mm [IQR: 15–22 mm]; *p* value: 0.047), and more severe residual MR (≥ MR III in 7.8 vs. 0%; *p* value: 0.008) compared with eligible counterparts (Table 5). They were also more symptomatic (NYHA III: 69.6 vs. 41.8%; *p* value: 0.045) and had higher NT-proBNP concentrations (4765 pg/mL [IQR: 2652–7230 pg/mL] vs. 2360 pg/mL [IQR: 1228–3500 pg/mL]; *p* value: 0.006) as well as lower hemoglobin levels (10.8 g/dL [IQR: 9.9–12.8 g/dL] vs. 12.7 g/dL [IQR: 11.6–13.6 g/dL]; *p* value: 0.007).

### Dynamic Reclassification of TTVI Eligibility From Baseline to Follow-Up: Frequent Changes Driven by Resolution of Severe TR After M-TEER

In a subgroup analysis restricted to patients with complete echocardiographic follow-up (Supplementary Table 4), baseline TTVI eligibility-like classification proved to be highly dynamic. Among the 54 patients with severe TR at baseline who would have been considered TTVI-eligible (i.e., absence of severe left ventricular dysfunction [LVEF <30%], severe pulmonary hypertension [sPAP >70 mmHg], or severe RV dysfunction [TAPSE <10 mm]), only 27 patients (50.0%) remained eligible at follow-up, while 19 (35.2%) no longer exhibited severe TR. Among the 51 patients with severe TR at baseline who were initially classified as TTVI-ineligible, reclassification was frequent: only 15.7% remained ineligible at follow-up, whereas the majority (66.7%) no longer had severe TR after M-TEER. In contrast, patients without severe TR at baseline were highly unlikely to develop severe TR at follow-up, with 93.7% remaining free of severe TR.

## Discussion

### Reframing Persistent TR Following M-TEER: TTVI Ineligibility as a Marker of Advanced Disease and Poor Prognosis

Our findings emphasize that persistent TR after M-TEER is not uniformly associated with poor outcomes. Prognosis depends largely on eligibility for subsequent TTVI, which in turn reflects the extent of cardiac damage. Ineligible patients accounted for nearly all TR-related excess mortality, whereas patients who remained eligible for TTVI showed survival comparable to those without severe TR. The prognostic impact of TTVI ineligibility persisted even after adjustment for age, biventricular function, and pulmonary pressure, indicating that the composite ineligibility profile—reflecting advanced cardiac remodeling—drives the excess mortality in this subgroup.

Collectively, these findings support the existence of an early interventional window, during which TR may still be amenable to transcatheter treatment before progression to end-stage heart failure precludes therapy. At the same time, our observation of comparable survival between patients without severe TR and those still eligible for TTVI (but not yet treated) raises an important question: *are we treating the right patients* *at the right time?* While this hypothesis is exploratory, it may help explain why neither TRILUMINATE nor TRISCEND II demonstrated a survival benefit despite marked symptomatic improvement.^6^, ^7^, ^8^

These hypothesis-generating data highlight the importance of identifying the optimal timing for intervention—since treatment that is performed too early (when patients are technically eligible but not yet hemodynamically compromised) may not yield a mortality benefit, whereas treatment that is delayed until ineligibility ensues may come too late to reverse cardiac and extracardiac damage. In line with this concept, a prior machine learning analysis using survival tree–based clustering identified 3 distinct risk categories among patients with severe TR and found that only those in the intermediate-risk group derived a statistically significant survival advantage from TTVI compared with medical therapy (HR for uncensored mortality 0.59 [95% CI: 0.37–0.91]; *p* value: 0.018).^13^

### Early Identification of Nonresponders: TR Persistence at 3 Months as a Prognostic Marker

In our cohort, 16.3% of patients had persistent severe TR at the 3-month follow-up. This finding is consistent with prior studies across more than 1200 patients, reporting persistent severe TR in approximately 13%–17% of patients after M-TEER, including 13% at 6 months and 17% at 12 months across independent cohorts.^1^^,^^14^^,^^15^ The similarity in these proportions may suggest that TR, which does not improve within the first 3 months after M-TEER, is unlikely to recover by 1 year.

This observation is clinically relevant, as improvement in TR severity has been associated with reduced mortality and amelioration of dyspnea symptoms,^2^^,^^15^ likely due to the unloading effect of MR correction on the pulmonary circulation (reflected by reductions in sPAP following M-TEER).^14^^,^^16^^,^^17^ Importantly, RV function and dimensions did not improve following M-TEER, suggesting that right-sided remodeling may represent a more advanced and less reversible stage of disease and thereby limit TR regression despite favorable left-sided and pulmonary hemodynamic changes.

Also consistent with previous studies, our regression analysis demonstrated that the extent of baseline right heart damage determines the severity of residual TR after M-TEER. Specifically, RV enlargement with papillary muscle displacement and tricuspid leaflet tethering (indicative of secondary ventricular TR), right atrial dilatation with subsequent annular enlargement and leaflet malcoaptation (indicative of secondary atrial TR), and TR severity itself were all associated with residual TR.

Atrial fibrillation, previously identified as a significant factor associated with severe TR at follow-up,^1^^,^^3^ also showed a strong association in univariable analysis but did not remain significant in the multivariable model. Notably, this apparent attenuation likely reflects the close interrelationship between atrial fibrillation and right atrial remodeling. Atrial fibrillation can be considered both a driver and a consequence of atrial dilatation and therefore acts as a surrogate marker of right atrial enlargement. When right atrial size was included in the multivariable model, the independent contribution of atrial fibrillation was attenuated, suggesting that its effect is largely mediated through structural right heart remodeling rather than representing an independent determinant of persistent TR. This finding is consistent with the known pathophysiology of secondary atrial TR and underscores the dominant role of right-sided structural changes over rhythm status per se.

Collectively, these data indicate that there is no added value in waiting for late recovery of severe TR; early residual TR appears to reflect underlying structural heart damage and is associated with persistent TR at long-term follow-up.

### Association of Female Sex With Persistent TR After M-TEER: Do We Need Sex-Specific Follow-Up Pathways?

Interestingly, female sex was significantly associated with the persistence of severe TR after M-TEER, even in the multivariable model. Attendance at follow-up echocardiography was comparable between men (255/512; 49.8%) and women (199/393; 50.6%) (*p* value: 0.857), indicating that no differential follow-up rates would explain this finding. Men and women also showed similar distributions of primary (34.6% in men vs. 33.1% in women) and secondary MR etiology (55.9% in men vs. 53.7% in women), with no statistically significant differences (*p* values: 0.690 and 0.560, respectively). Furthermore, the prevalence of severe TR at baseline (grade ≥3) did not differ between men and women (21.3% vs. 25.4%, *p* value: 0.150). However, at follow-up, women exhibited a significantly higher rate of residual severe TR compared with men (20.6% vs. 12.9%, *p* value: 0.039).

A plausible mechanistic explanation for this sex-specific difference is that women may more often exhibit an atrial functional TR phenotype, whereas men may more often present with ventricular TR related to ischemic heart disease and left ventricular dysfunction. In a multicenter TTVI cohort including 702 patients, secondary atrial TR was more frequent in women than in men (41.7% vs. 24.4%), while secondary ventricular TR was more common in men (64.6% vs. 50.0%).^18^ Men in that cohort also more often had coronary artery disease (52.9% vs. 35.5%) and lower LVEF (51.2% vs. 55.6%). Similarly, data from the Transcatheter Tricuspid Valve Therapies (TriValve) registry revealed lower LVEF in men than in women undergoing TTVI (46.3% vs. 53.8%) and described more advanced ventricular disease in men.^19^ This framework may also help explain the apparently paradoxical association between higher baseline LVEF and persistent TR in our multivariable model: in the setting of M-TEER, preserved LVEF may identify patients with a more atrial/right-sided remodeling phenotype, in whom TR is less driven by left ventricular dysfunction and therefore less likely to regress despite successful M-TEER.

Moreover, several recent studies demonstrate that women with valvular disease experience both delayed diagnosis and referral as well as distinct structural remodeling patterns.^20^, ^21^, ^22^ Women exhibit greater left atrial dilation, more atrial functional MR, and more extensive annular remodeling than men, even at similar quantitative MR thresholds. This is partly explained by guideline definitions based on absolute rather than indexed left ventricular and left atrial cutoffs, which tend to underestimate disease severity in women. The resulting later presentation and more advanced atrial disease in women may predispose to a more atrial TR phenotype, characterized by limited capacity for reverse remodeling after M-TEER. However, given the relatively small number of events and the wide CIs, this finding should be interpreted with caution and may reflect residual confounding or model instability rather than a robust independent effect. Confirmation in larger cohorts is therefore warranted.

Because women have consistently been shown to experience underdiagnosis, undertreatment, and treatment delays across various cardiovascular diseases,^23^ our results underscore the need for heightened awareness and tailored management strategies in women with MR—particularly those with concomitant TR, who may be at increased risk of persistent right-sided dysfunction after M-TEER.

### Preventing Advanced TR: Why Early Recognition Matters in the Absence of Effective Therapies

This study is retrospective, and patients who would today qualify for staged TTVI for persistent TR after M-TEER were not treated with this option at the time. Furthermore, for patients deemed ineligible for TTVI, we still lack effective therapeutic alternatives. Management of right-sided heart failure remains largely limited to diuretics, as no neurohormonal modulation comparable to left-sided heart failure therapy is currently available.^24^ The markedly worse prognosis observed in TTVI-ineligible patients—with a 2-year survival of only 39.5% (95% CI: 22.8%–68.4%)—highlights that these patients were likely diagnosed or treated too late. As long as no interventional or pharmacological treatment options exist for this group, preventive strategies and earlier recognition of disease progression are essential to minimize the number of patients reaching such an advanced, treatment-limited stage.

### Baseline TTVI Eligibility Is Dynamic and Requires Reassessment After M-TEER

Beyond its prognostic implications, our data demonstrate that TTVI eligibility is not a static construct but rather evolves following M-TEER. While baseline severe TR identifies a high-risk population, eligibility-like classification at baseline proved to be highly unstable, with substantial reclassification observed after intervention—particularly among patients initially considered TTVI-ineligible, the majority of whom no longer fulfilling ineligibility criteria at follow-up (most commonly due to resolution of severe TR). These findings indicate that baseline guideline-derived, eligibility-like criteria reflect a preinterventional hemodynamic state rather than a fixed disease stage and may therefore overestimate disease severity and ineligibility. From a clinical perspective, this supports a staged approach in which baseline severe TR prompts closer surveillance, whereas definitive assessment of candidacy for TTVI should be deferred to follow-up after M-TEER. Notably, patients without severe TR at baseline were highly unlikely to develop severe TR during follow-up, underscoring that baseline TR severity remains useful for risk stratification but not for upfront treatment planning.

Conceptually, guideline-based TTVI eligibility criteria may be interpreted as a composite, clinically pragmatic “multidimensional biomarker” integrating left ventricular function, pulmonary pressure, and RV performance. This integrative approach may better capture advanced disease stages than single parameters such as pulmonary hypertension, RV function, or RV to pulmonary artery coupling alone.^25^, ^26^, ^27^ Importantly, this concept is supported by our exploratory analysis of RV to pulmonary artery coupling: patients deemed TTVI-ineligible exhibited significantly impaired TAPSE/sPAP ratios compared with eligible patients (0.267 mm/mmHg [95% CI: 0.181–0.355 mm/mmHg] vs. 0.386 mm/mmHg [95% CI: 0.295–0.485 mm/mmHg]; Table 5), indicating advanced RV to pulmonary artery uncoupling. These findings suggest that guideline-based eligibility criteria capture a multidimensional state of cardiac decompensation that aligns with established physiological markers while providing a clinically applicable framework beyond isolated parameters. Importantly, patients classified as TTVI-eligible in this study did not undergo TTVI. Thus, the observed differences in outcomes should be interpreted as reflecting underlying disease severity rather than a treatment effect.

### Limitations

This study has several limitations that should be considered when interpreting the results.

First, the analysis is retrospective, and although data were prospectively collected, residual confounding cannot be excluded. In addition, the number of patients classified as TTVI-ineligible was relatively small, which may limit the precision of survival estimates and warrants confirmation in larger cohorts. Furthermore, information on medical therapy from baseline to follow-up (e.g., diuretic adjustment or heart failure therapies such as renin-angiotensin system inhibitors and sodium-glucose cotransporter-2 inhibitors) was not systematically available and may have influenced both TR persistence and clinical outcomes.

Second, only half of the cohort attended follow-up echocardiography (454/905; 50.2%). This is likely explained by the advanced age (median: 79.7 years) and frailty of the study population as well as logistical challenges related to referral patterns to tertiary centers, including long travel distances. In addition, a proportion of patients may have died or were clinically unable to return for follow-up. Importantly, patients with no attendance of follow-up echocardiography had significantly higher mortality, introducing a risk of selection bias, as follow-up findings may disproportionately reflect outcomes in healthier survivors. Furthermore, although vital status was comprehensively ascertained through the German Civil Registry, information on cause of death was not available; therefore, analyses were limited to all-cause mortality without differentiation between cardiovascular and non-cardiovascular causes.

Third, guideline-based TTVI eligibility criteria were applied retrospectively, and patients who would today qualify for staged TTVI were not treated with this option during the study period, reflecting historical practice rather than contemporary care pathways. Future studies using advanced causal inference approaches, such as propensity score matching, are warranted to disentangle the potential treatment effect of TTVI from its prognostic role in large real-world registries of M-TEER patients with persistent TR stratified according to guideline-based TTVI eligibility criteria.

Fourth, echocardiographic assessment was performed during routine clinical practice at 2 centers, which may introduce interobserver variability despite adherence to guideline-defined measurement standards. Moreover, while we applied guideline-based thresholds for RV dysfunction (TAPSE <10 mm), this represents a relatively advanced stage of impairment. More conservative thresholds (e.g., TAPSE <14 mm) may identify earlier stages of RV dysfunction and could influence eligibility classification, potentially excluding an even larger proportion of patients from TTVI eligibility after M-TEER.

Fifth, although we evaluated anatomical and hemodynamic criteria for TTVI eligibility, we could not incorporate more nuanced anatomical features (e.g., leaflet graspability, coaptation gap geometry, or annular dynamics) that may influence real-world TTVI decision-making.

Finally, the study population was predominantly elderly and multimorbid, limiting generalizability to younger or lower-risk populations. Furthermore, no formal adjustment for multiple comparisons was performed; therefore, the results should be interpreted as exploratory and hypothesis-generating. Despite these limitations, the dual-center design, large cohort, and complete survival follow-up strengthen the robustness of our findings.

## Conclusion

In this dual-center cohort of 905 patients undergoing M-TEER, severe TR persisted in approximately one-sixth of patients at 3-month follow-up. Early persistent TR was strongly associated with structural right heart remodeling, advanced preprocedural TR severity, and female sex. Importantly, prognosis among patients with persistent TR was not uniform: excess mortality was almost entirely confined to those who met contemporary exclusion criteria for TTVI. In contrast, patients with persistent TR who remained TTVI eligible demonstrated survival comparable to those without severe TR.

These findings highlight persistent TR after M-TEER as an early marker of right-sided disease progression and underscore the importance of structured follow-up to promptly identify patients who may benefit from staged TTVI. Moreover, the markedly poor outcomes of TTVI-ineligible patients emphasize that once advanced right-sided or pulmonary vascular disease develops, therapeutic options remain limited. Early recognition, timely referral, and consideration of staged TTVI within an interventional “window of opportunity” may help prevent progression to end-stage disease. Future prospective studies are warranted to define optimal timing, refine selection criteria, and evaluate whether early staged TTVI can improve clinical outcomes in this growing patient population.

## Data Sharing Statement

The data underlying this article will be shared on reasonable request to the corresponding author. All requests for raw and analyzed data and related materials will be reviewed by the Ethics Committee at Technical University of Munich, Germany. Any data and materials that can be shared will be released via a Material Transfer Agreement.

## Funding

M. Lachmann has received funding from the Technical University of Munich (Clinician Scientist Grant), from the Else Kröner-Fresenius Foundation (Clinician Scientist Grant), the German Center for Cardiovascular Research (DZHK; Postdoc Start-up Grant on Advancing Digital Aspects), and the German Heart Foundation (“Machine learning in severe mitral regurgitation”). V. Fortmeier has received funding from Ruhr University Bochum (Female Clinician Scientist Grant). A. Hesse received funding from the German Cardiac Society (DGK; Otto Hess Doctoral Scholarship). T. Trenkwalder received funding from the Else Kröner-Fresenius Foundation (Clinician Scientist Grant) and the German Heart Foundation.

## Disclosure Statement

The authors report no conflict of interest.
