# Supplementary material for: Persistent Severe Tricuspid Regurgitation After Mitral Transcatheter Edge-to-Edge Repair: Prognostic Implications of Guideline-Based Eligibility for Transcatheter Tricuspid Valve Intervention
Source: Struct Heart. 2026 May 22;10(7):101051. doi: 10.1016/j.shj.2026.101051 (PMC13316627; doi:10.1016/j.shj.2026.101051)
Supplement: Supplementary Material [file mmc1.pdf]

Supplemental Material

Supplementary Figure 1: Survival after M-TEER in patients with and without follow-up echocardiography.

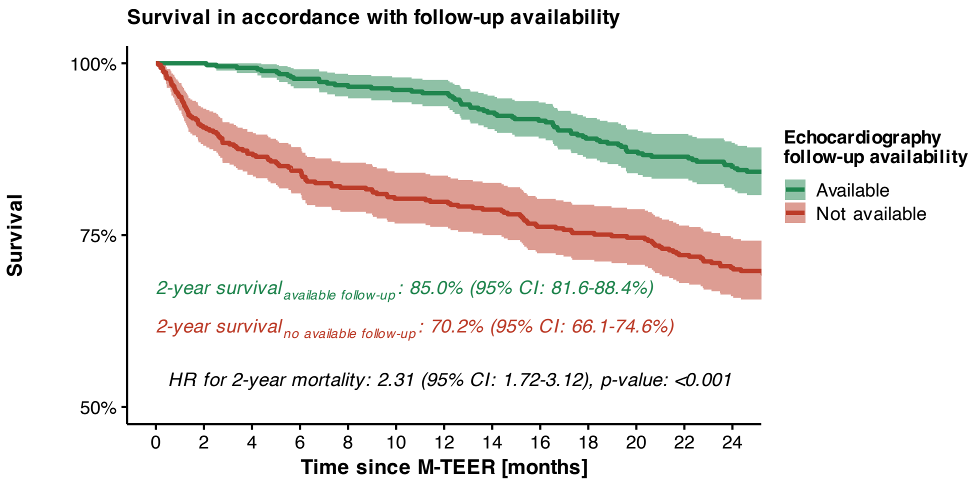

Number at risk

Available	454	449	441	429	416	411	409	393	388	374	363	358	349
Not available	451	406	387	374	361	354	352	347	336	330	325	314	303

Supplementary Figure 2: Survival in accordance with TTV eligibility (landmark analysis).

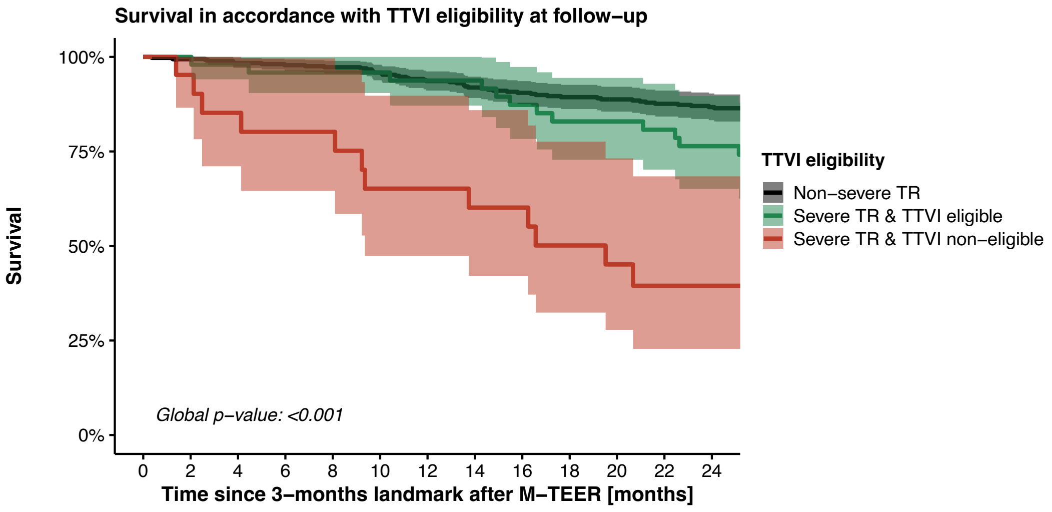

Number at risk

	0	2	4	6	8	10	12	14	16	18	20	22	24
Non−severe TR	373	366	359	352	349	341	332	326	318	311	307	301	293
Severe TR & TTVI eligible	49	49	47	45	45	45	44	44	40	38	38	37	35
Severe TR & TTVI non−eligible	21	19	17	16	16	13	13	12	12	10	9	7	7

Supplementary Table 1: Implanted Devices.

Device	No. (%)
**MitraClip, 1st generation, No. (%)**	104 (11.5%)
**MitraClip, 2nd generation (NT), No. (%)**	141 (15.6%)
**MitraClip, 3rd generation (NTR/XTR), No. (%)**	207 (22.9%)
**MitraClip, 4th generation (NTW/XTW), No. (%)**	211 (23.3%)
**PASCAL, 1st generation, No. (%)**	126 (13.9%)
**PASCAL Ace, No. (%)**	116 (12.8%)

Categorical variables are presented as counts and frequencies.

Supplementary Table 2: Demographic and Clinical Baseline Characteristics of the Study Population According to Echocardiographic Follow-up Attendance.

	Study population			
	All (*n* = 905 patients)	Available follow-up (*n* = 454 patients)	No follow-up (*n* =451 patients)	*p*-value
**Age, years**	79.7 (74.2-83.4)	79.6 (74.5-83.4)	79.7 (73.8-83.3)	0.835
**Female, No. (%)**	393 (43.4%)	199 (43.8%)	194 (43.0%)	0.857
**BMI, kg/m^2**	25.0 (22.5-28.2)	25.0 (22.6-28.4)	25.0 (22.5-28.1)	0.616
**Arterial hypertension, No. (%)**	754 (83.3%)	388 (85.5%)	366 (81.2%)	0.099
**Diabetes mellitus, No. (%)**	231 (25.5%)	119 (26.2%)	112 (24.8%)	0.690
**History of CAD, No. (%)**	555 (61.3%)	271 (59.7%)	284 (63.0%)	0.345
**History of COPD, No. (%)**	143 (15.8%)	68 (15.0%)	75 (16.6%)	0.555
**History of atrial fibrillation, No. (%)**	657 (72.6%)	327 (72.0%)	330 (73.2%)	0.756
**NYHA ≤II, No. (%)**	176 (19.4%)	89 (19.6%)	87 (19.3%)	0.972
**NYHA III, No. (%)**	609 (67.3%)	324 (71.4%)	285 (63.2%)	0.011
**NYHA IV, No. (%)**	120 (13.3%)	41 (9.0%)	79 (17.5%)	<0.001
**EuroSCORE II, %**	5.13 (3.26-8.54)	4.67 (2.96-7.90)	5.58 (3.54-9.73)	<0.001
**eGFR, mL/min**	48 (35-62)	50 (37-65)	46 (34-60)	0.005
**NT-proBNP, pg/mL**	2,810 (1,293-6,545)	2,555 (1,160-5,510)	3,350 (1,525-7,628)	0.001
**Hemoglobin, g/dL**	12.6 (11.1-13.7)	12.8 (11.4-13.9)	12.3 (10.8-13.5)	<0.001
**Dialysis, No. (%)**	36 (4.0%)	9 (2.0%)	27 (6.0%)	0.004
**Etiology**				
**Primary, No. (%)**	307 (33.9%)	148 (32.6%)	159 (35.3%)	0.439
**Secondary, No. (%)**	497 (54.9%)	256 (56.4%)	241 (53.4%)	0.409
**Mixed, No. (%)**	101 (11.2%)	50 (11.0%)	51 (11.3%)	0.972

Categorical data are presented as counts and frequencies (%), while continuous data are expressed as median and interquartile range.

Abbreviations as in Tables 1 and 2.

Supplementary Table 3: Echocardiographic Baseline Characteristics of the Study Population According to Echocardiographic Follow-up Attendance.

	Study population			
	**All** (*n* = 905 patients)	**Available follow-up** (*n* = 454 patients)	**No follow-up** (*n* =451 patients)	***p*-value**
**LVEF, %**	48 (33-58)	50 (35-58)	46 (31-58)	0.035
**LVEDD, mm**	57 (50-63)	57 (50-63)	56 (50-63)	0.808
**LVESD, mm**	41 (34-51)	40 (33-50)	42 (34-53)	0.111
**LVEDV, mL**	144 (95-196)	142 (95-193)	144 (95-196)	0.545
**LVESV, mL**	80 (44-128)	71 (43-127)	86 (45-133)	0.099
**MV EROA, cm²**	0.3 (0.2-0.4)	0.3 (0.2-0.4)	0.3 (0.2-0.4)	0.591
**MR vena contract width, cm**	0.7 (0.6-1.0)	0.7 (0.6-1.0)	0.8 (0.6-1.0)	0.164
**MV regurgitation volume, mL**	47 (35-66)	49 (37-68)	47 (34-63)	0.161
**LA volume, mL**	125 (96-169)	121 (88-168)	130 (100-169)	0.169
**sPAP, mmHg**	46 (36-58)	45 (34-56)	47 (37-60)	0.017
**Right midventricular diameter, mm**	31 (27-35)	30 (26-34)	32 (27-36)	0.081
**TAPSE, mm**	17 (14-20)	17 (14-21)	17 (13-20)	0.156
**RA area, cm²**	26 (20-31)	25 (19-30)	26 (21-32)	0.038
**MR II and II+/IV°, No. (%)**	485 (53.6%)	183 (40.3%)	302 (67.0%)	<0.001
**MR III and III+/IV°, No. (%)**	246 (27.2%)	171 (37.7%)	75 (16.6%)	<0.001
**MR IV/IV°, No. (%)**	174 (19.2%)	100 (22.0%)	74 (16.4%)	0.039
**TR ≥III/IV°, No. (%)**	209 (23.1%)	105 (23.1%)	104 (23.1%)	1.0

Categorical data are presented as counts and frequencies (%), while continuous data are expressed as median and interquartile range.

Abbreviations as in Tables 1 and 2.

Supplementary Table 4: Re-Classification of Tricuspid Regurgitation Severity and Eligibility Status.

Only patients with available follow-up echocardiographic assessment (n = 454) were included in this analysis. Percentages reflect the proportion of patients within each baseline category transitioning to each follow-up category.

	No severe TR at follow-up	Severe TR, eligible at follow-up	Severe TR, ineligible at follow-up	
**No severe TR at baseline**	327 (93.7%)	15 (4.3%)	7 (2.0%)	349 (76.9%)
**Severe TR, eligible-like at baseline**	19 (35.2%)	27 (50.0%)	8 (14.8%)	54 (11.9%)
**Severe TR, ineligible-like at baseline**	34 (66.7%)	9 (17.6%)	8 (15.7%)	51 (11.2%)
**Total**	380	51	23	454 (100%)

Abbreviations as in Tables 1 and 2.
